# 3D Printed Bigel: A Novel Delivery System for Cannabidiol-Rich Hemp Extract

**DOI:** 10.3390/gels10120770

**Published:** 2024-11-26

**Authors:** Anna Gościniak, Filip Kocaj, Anna Stasiłowicz-Krzemień, Marcin Szymański, Tomasz M. Karpiński, Judyta Cielecka-Piontek

**Affiliations:** 1 Department of Pharmacognosy and Biomaterials, Poznan University of Medical Sciences, Rokietnicka 3, 60-806 Poznan, Poland; agosciniak@ump.edu.pl (A.G.); 83122@student.ump.edu.pl (F.K.); astasilowicz@ump.edu.pl (A.S.-K.); 2 Center for Advanced Technologies, Adam Mickiewicz University in Poznań, Uniwersytetu Poznańskiego 10, 61-614 Poznan, Poland; marcin.szymanski@amu.edu.pl; 3 Department of Medical Microbiology, Medical Faculty, Poznan University of Medical Sciences, Rokietnicka 10, 60-806 Poznan, Poland; tkarpin@ump.edu.pl

**Keywords:** bigels, cannabidiol, cannabidiolic acid, cannabis

## Abstract

The therapeutic potential of *Cannabis sativa* L. extract has gained significant attention due to its diverse medical applications. Sublingual administration remains a common delivery method of cannabinoids; however, challenges often arise due to the inconvenient form of the extract and its taste. To address these issues, a novel bigel formulation was developed, combining water and oil phases to enhance stability and bioavailability. This formulation incorporates a cannabidiol-rich hemp extract, hyaluronic acid for its moisturizing properties, and a taste-masking agent to improve patient compliance and comfort. Using a standardized hemp extract rich in cannabinoids and a well-characterized terpene profile, the printability of the bigels was evaluated through 3D printing technology. A printout with known cannabidiol (CBD) and cannabidiolic acid (CBDA) content of 11.613 mg ± 0.192 of CBD and 4.732 mg ± 0.280 of CBDA in the printout was obtained. In addition, the release profile of CBD and CBDA was evaluated to determine the delivery efficiency of the active ingredient—dissolved active ingredient levels ranged from 74.84% ± 0.50 to 80.87% ± 3.20 for CBD and from 80.84 ± 1.33 to 98.31 ± 1.70 for CBDA depending on the formulation. Rheological studies were conducted to evaluate the viscosity of the bigels under varying temperature conditions, ensuring their stability and usability. Findings suggest that this 3D-printed bigel formulation could significantly enhance the delivery of cannabis extracts, offering a more convenient and effective therapeutic option for patients. This research underscores the importance of innovation in cannabinoid therapies and paves the way for further advancements in personalized medicine.

## 1. Introduction

Bigels are innovative delivery systems that combine the structural properties of hydrophilic and hydrophobic gels, forming a biphasic network that can simultaneously encapsulate and deliver both water-soluble and fat-soluble active compounds [1]. This dual-phase system makes bigels highly versatile for a wide range of applications, particularly in pharmaceuticals and cosmetics, where controlled release and bioavailability of both types of substances are crucial [2,3,4]. The production process typically involves emulsifying an aqueous phase (hydrogel) with an oil phase (organogel), requiring precise control over the stability of the emulsion to achieve the desired consistency, rheological properties, and functional performance. In the study by Kurapkienė et al. [5], a bigel structured with collagen, monoglycerides, and diglycerides significantly improved the stability of curcumin, a compound known for its antioxidant and anti-inflammatory properties but that is typically unstable and poorly bioavailable. The bigel system maintained over 97% of curcumin’s content during both the manufacturing process and in vitro digestion, effectively addressing curcumin’s degradation and solubility issues.

The search for alternatives to traditional pharmaceuticals has intensified due to several limitations associated with conventional drug therapies. Many classic medications can cause adverse side effects, lead to tolerance, or have limited efficacy, particularly in managing chronic conditions such as pain, anxiety, and inflammatory diseases. Furthermore, issues such as drug resistance, especially in the case of antibiotics, and the environmental impact of pharmaceutical residues have driven research toward more sustainable and biocompatible options. Plant-based compounds, like cannabinoids from cannabis, offer a promising alternative as they possess therapeutic properties with generally favorable safety profiles and lower risk of severe side effects. Additionally, alternative delivery systems, such as bigels, enhance the bioavailability and targeted delivery of these compounds, potentially improving treatment outcomes while reducing systemic exposure and minimizing side effects. This push towards innovative, naturally derived treatments reflects a growing demand for safer, more effective, and patient-centric therapeutic options.

Cannabidiol (CBD)-rich cannabis extracts have garnered attention for their therapeutic potential in treating various medical conditions. These extracts are high in CBD and low in Δ9-tetrahydrocannabinol (Δ9-THC), offering non-psychoactive treatment options. They are particularly effective in managing epilepsy, cancer-related symptoms, chronic pain, and anxiety disorders [6,7,8]. Research also supports the use of CBD-rich extracts in veterinary medicine for pain and anxiety in animals [9]. As research progresses, the potential applications of CBD in medicine continue to expand. Mucosal and buccal delivery systems for cannabis represent an innovative approach to administering cannabinoids, offering several advantages over traditional methods [10]. These systems leverage the rich vascularization of the oral mucosa to facilitate rapid absorption of cannabinoids into the bloodstream, thereby enhancing bioavailability and providing quicker onset of effects compared to oral ingestion [11,12]. This route is particularly beneficial for patients requiring immediate relief from symptoms, such as pain or nausea, as it bypasses the gastrointestinal tract and first-pass metabolism in the liver, which can significantly reduce the effective dose of cannabinoids [13]. One of the most notable examples of a mucosal delivery system is Sativex^®^, an oromucosal spray that contains both THC and CBD. This formulation has been shown to provide effective symptom relief for conditions such as multiple sclerosis and cancer-related pain [11,14]. The pharmacokinetics of oromucosal delivery systems indicate that cannabinoids can achieve therapeutic plasma concentrations rapidly, making this method particularly appealing for patients who may experience acute symptoms [11].

Buccal delivery systems, such as lozenges or films, also present a viable alternative for cannabinoid administration. These formulations can be designed to dissolve slowly in the mouth, allowing for sustained release of cannabinoids and prolonged therapeutic effects. Research has demonstrated that buccal delivery can enhance the absorption of cannabinoids, leading to improved patient compliance and satisfaction due to the ease of use and discreet nature of the administration. For instance, Trokie^®^ lozenges have been developed specifically for the buccal delivery of cannabis extracts, providing a standardized dosage form that is both effective and user-friendly [15].

An additional benefit of hemp-based bigel antibacterial properties is that it has been increasingly recognized in recent studies. For instance, hemp extract has been shown to inhibit the growth of various bacterial strains [16,17]. This adds a new dimension to the potential of application-based bigels, particularly in the field of oral care products.

Moreover, the application of 3D printing technology in the development of personalized dosage forms has become a promising area of research. Modern technologies that modify the properties of existing materials and active substances have the potential to significantly improve medical treatments [18,19]. The integration of 3D printing technology into personalized medicine represents a transformative advancement in pharmaceutical manufacturing and patient care. This technology allows for the customization of drug formulations tailored to individual patient needs, which is particularly beneficial in addressing the variability in drug response among patients. The ability to produce specific dosages, shapes, and release profiles through 3D printing enhances the precision of drug delivery systems, thereby improving therapeutic outcomes and patient compliance [20,21]. The adaptability of bigels, capable of transitioning between liquid and semi-solid states, makes them ideal candidates for extrusion-based 3D printing processes. This approach allows for precise control over drug loading, release kinetics, and dosage customization. By adjusting the composition and structure of the bigel, it is possible to tailor the release profile of active ingredients to meet individual patient needs, offering a more personalized therapeutic approach. The flexibility of 3D printing also facilitates the creation of size, making it possible to design formulations that enhance patient adherence and satisfaction with personalized doses.

The aim of the present study was to develop a novel bigel-based drug delivery system containing hemp extract, in which the oil phase consists of hemp oil and the aqueous phase incorporates hyaluronic acid, a moisturizing agent. Hyaluronic acid was included to counteract the potential drying effects of hemp extract, particularly in oral applications. Menthol was also added to enhance the formulation by improving the permeability and providing a cooling sensation [22,23]. The innovation of this approach lies in the combination of these ingredients within a bigel system, which allows for the controlled release of bioactive compounds. The system was designed as single-dose printed dosages, enabling precise control over drug loading and release. This customizable platform offers a balanced and therapeutic formulation, which can be tailored to meet individual patient needs, ensuring more effective and personalized cannabinoid delivery. By using 3D printing technology, the study also introduces a novel way to create personalized dosages, enhancing patient compliance and satisfaction while optimizing therapeutic outcomes.

## 2. Results and Discussion

### 2.1. Choosing the Strain with the Highest Content of Cannabinoids

Seven cannabis strains were extracted using supercritical carbon dioxide (scCO_2_), and the resulting methanol-solubilized extract was analyzed through HPLC. Significant differences in total cannabinoid content were observed among the strains (Table 1). The content of cannabinoids, including CBD, CBDA, cannabigerol (CBG), cannabinol (CBN), cannabigerolic acid (CBGA), delta-9-tetrahydrocannabinol (Δ9-THC), delta-8-tetrahydrocannabinol (Δ8-THC), cannabichromene (CBC), and tetrahydrocannabinolic acid (THCA), was evaluated. The Black Widow strain exhibited the highest levels of all determined cannabinoids and was selected for further formulation studies. However, it should be noted that the extraction applied proved effective for each of the varieties analyzed. The use of scCO_2_ extraction is one of the best methods for obtaining extracts rich in cannabinoid compounds while maintaining the principles of ‘green chemistry’ [24]. The variability in cannabinoid content across different cannabis strains can be attributed to several factors, including genetic variation, cultivation practices, and environmental influences [25,26]. The choice of variety has a direct effect on the cannabinoid content of the extract obtained from cannabis [27]. Depending on the desired clinical effect, varieties with different cannabinoid contents are used. The main ones are CBD and THC. In the study we conducted, all strains were high in CBD and low in THC. CBD has no psychoactive properties, and its use is not restricted by law like THC. At the same time, it can have beneficial health effects such as calming, improving sleep, antioxidant or anti-inflammatory aspects [28].

Many publications have compared cannabis strains to assess their cannabinoid content. In the study by Glivar et al. [29], 15 different hemp strains grown in Slovenia were compared, including a comparison of the two seed years. Significant differences in cannabinoid content between strains were identified for 13 analyzed cannabinoids. The uppermost inflorescences were analyzed, revealing the highest total CBD content (>5% *w*/*w*) in the bracts of Antal (9.580% ± 1.287 in 2017, 6.159% in 2018), Carmagnola (7.069% ± 3.339 in 2017, 5.726% in 2018), Helena (5.873% ± 1.816 in 2017, 8.108% in 2018), and Tiborszallasi (4.666% ± 1.931 in 2017, 4.876% in 2018).

Furthermore, the study by Janatova et al. [30] evaluated the performance of various strains in terms of biomass yield and cannabinoid production. This research identified strains with high yields and stable production of therapeutic cannabinoids, such as ‘Nurse Jackie’ and ‘Jilly Bean’, highlighting the importance of selecting specific strains to meet medical needs. Average Δ9-THC levels for each strain across six growing cycles ranged from 15.69 ± 2.6% to 19.31 ± 2.47% (*w*/*w*), while average CBD levels ranged from 0.45 ± 0.1% to 0.57 ± 0.08% (*w*/*w*). Another significant study, conducted by Abdollahi et al. [31], demonstrated that cannabinoid levels fluctuate during various growth stages and are influenced by regional environmental factors. This emphasizes the importance of optimal strain selection and precise timing for cultivation. CBD content across all growth stages of the strains studied in three regions ranged from 0% to 1.39%. Our results are similar to other studies that have investigated CBD-rich strains, but it should be remembered that the type of extraction used is also important.

### 2.2. Characteristics of the Extract Chosen for the Formulation

#### 2.2.1. HPLC Analysis

Cannabinoids are chemical compounds found naturally in cannabis that affect the human endocannabinoid system [7]. In the cannabis plant, they are found in acidic forms, such as CBDA, THCA (tetrahydrocannabinolic acid), and CBGA (cannabigerolic acid), which are converted to active forms, such as CBD, THC, or CBG, when exposed to heat (decarboxylation process). The intra-entourage effect—referring to the synergistic interaction between cannabinoids—further increases the therapeutic potential of these compounds. Cannabinoids influence each other, which means that their combination can potentiate or diminish certain effects (the so-called synergistic or entourage effect).

For the selected extract (from the Black Widow strain), the extraction was repeated, and the winterized extract was concentrated and characterized for use in formulation development. Table 2 shows the cannabinoids’ content in 1 mL of the final extract. The procedures conducted yielded an extract with CBD content suitable for creating a precise dosage formulation. The dosage regimen of CBD varies greatly depending on the expected therapeutic effect and the route of administration. For administration via oral mucosal absorption, e.g., sublingual administration, the most commonly studied doses range from 15 to 70 mg/day [32,33,34]. In the study by Baratta et al. [35], an oil extract was obtained with cannabinoid concentrations as follows: CBD 12.092 mg/mL, THC 6.971 mg/mL, CBDA 0.631 mg/mL, THCA 0.036 mg/mL, and CBN 0.450 mg/mL, with the note that a THC-rich cannabis strain was used. Full-spectrum cannabis extracts were also used in a study by Villate et al. [36] to obtain PLGA nanoparticles also for oral administration. In some studies, such as Taha et al. [37] where buccal film formulations were proposed, isolated CBD was used. There, CBD constituted 10% (*w*/*w*) of the film.

#### 2.2.2. GC–MS Analysis

The results of the GC–MS analysis indicate the presence of various compounds in the sample (Table 3). The largest percentage (93.848%) is CBD, which is the main active compound. Other important compounds are caryophyllene (2.548%) and humulene (0.826%), terpenes known for their anti-inflammatory and analgesic properties. Rarer compounds are also present, such as cis-5,8,11,14,17-eicosapentaenoic acid (EPA) (0.324%), which is a polyunsaturated fatty acid with anti-inflammatory effects [38], which suggests that the fatty compounds were adsorbed by supercritical CO_2_ extraction. The winterization process was not fully effective, resulting in some fats and waxes remaining in the final extract. The overall composition suggests a CBD-rich sample with additional terpenes and other bioactive compounds that may work together as part of an entourage effect, increasing the therapeutic potential of the sample. In addition to cannabinoids, terpenes are the key active ingredients in hemp extract. The interaction of all components, called the entourage effect, is responsible for the strain’s unique effects. Among the notable terpenes identified, caryophyllene and humulene are significant due to their anti-inflammatory and analgesic properties [39,40,41]. Caryophyllene, a bicyclic sesquiterpene, has been documented to function as a selective agonist of the CB2 receptor, part of the endocannabinoid system [42,43]. Its activation of CB2 receptors leads to the modulation of immune responses, making it effective in reducing inflammation. Additionally, caryophyllene has been shown to provide neuroprotective benefits and assist in managing chronic pain [44]. Similarly, humulene, a monocyclic sesquiterpene, is recognized for its potential to reduce inflammation, and it may complement together with caryophyllene the effects through synergistic mechanisms [45,46]. Research indicates that humulene inhibits the production of pro-inflammatory cytokines, such as TNF-α and IL-1β, in response to inflammatory stimuli [46,47]. The sample also contains other sesquiterpenes, including α-acorenol and β-guaiene, which are less studied but exhibit promising antimicrobial properties [48,49,50,51]. Sesquiterpenes, in general, are known for their broad-spectrum biological activities, particularly in defending plants against microbial pathogens [52]. The antimicrobial potential of α-acorenol and β-guaiene suggests that these compounds may contribute to the extract’s ability to combat bacterial and fungal infections, making the extract potentially useful for topical applications. The terpene profile of cannabis varies significantly depending on the strain, as confirmed by analyses using gas chromatography–mass spectrometry. A study by Ahmed et al. [53] identified six major terpenes—α-pinene, β-pinene, myrcene, limonene, β-caryophyllene, and α-humulene—in various cannabis flower samples, with concentrations differing across strains. A study by Kaur et al. [54] documented the presence of over 146 terpenes and terpenoids in cannabis, emphasizing the dominance of α-pinene and limonene in many strains. This chemical diversity and the proportions of these compounds contribute to the distinct aromas and potential therapeutic effects characteristic of individual strains.

#### 2.2.3. Results of the Analysis of Antimicrobial Properties

In the analysis of antimicrobial activity, the results obtained demonstrated varying antimicrobial activity of the hemp extract against pathogens associated with skin infections, oral health, and respiratory diseases (Table 4). Particularly strong effects were observed against *Streptococcus mutans*, a key contributor to dental caries. Moderate effectiveness was seen against pathogens like *Pseudomonas aeruginosa* and *Candida albicans*, which frequently cause infections in immunocompromised individuals, as well as *Porphyromonas gingivalis* and *Fusobacterium nucleatum*, both associated with periodontal diseases. The lowest activity was observed against *Staphylococcus aureus*, a bacterium responsible for skin and hospital-acquired infections, suggesting that the extract may have limited applications in treating these types of infections. These results highlight the potential of hemp extract in preventing and treating oral diseases. Given this fact, the proposed formulation has the potential not only to deliver CBD and other cannabinoids but also to provide antibacterial effects in the oral environment. Ali et al. reported that cannabis seed oil exhibited significant antibacterial effects, with inhibition zones ranging from 21 to 28 mm against *Bacillus subtilis* and *Staphylococcus aureus*, and moderate activity against *Escherichia coli* and *Pseudomonas aeruginosa* [55]. Further investigations into the specific components responsible for this activity have identified cannabinoids, terpenes, and phenolic compounds as significant contributors. For example, Malikova highlighted that cannabinoids such as THC and CBD have shown antibacterial effects, for example, against *Staphylococcus aureus* [56].

### 2.3. Comparison of Properties of Bigels

#### 2.3.1. Composition and Stability Assessment of Bigels

Formulations were obtained with the compositions shown in Table 5.

**Figure 1:**
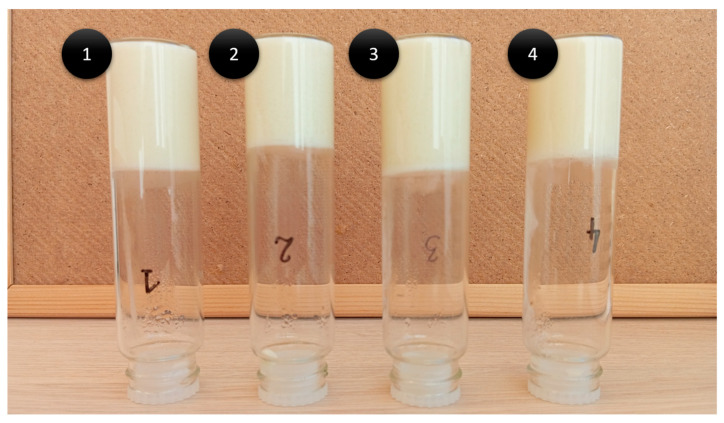
Results of the inverted tube test (numbers 1–4 correspond to bigels 1–4).

The preparations showed a creamy yellow color and uniform consistency. In the inverted tube test, which is used to assess the stability and gelation of the formulations, their stability was confirmed (Figure 1). This test showed that all formulations retained their integrity without any flow under gravitational conditions, indicating good stability over time. In addition, the formulations exhibited a distinct menthol aroma alongside the characteristic smell of hemp.

Similar results regarding the stability of bigel formulations containing plant extracts were reported by Sotirova et al. [57], who confirmed that a properly selected composition minimizes phase separation, enabling the creation of a uniform formulation containing St. John’s Wort Extract. To date, efforts to maintain the stability of bigels containing plant extracts have been successful, including systems for delivering active compounds from Centella asiatica, as well as bigels containing 1,4-naphthoquinones [58,59]. Bigels also show potential as delivery systems, as highlighted in the review by Zampouni et al. [60] Additionally, Chen et al. [61] developed 3D-printed bigel systems based on beeswax oleogels and HPMC (hydroxypropyl methyl cellulose) hydrogels, prepared using an in situ gelation method. Their study also confirmed that bigels could be 3D printed effectively. Another important aspect is the organoleptic analysis of formulations. The cooling sensation produced by menthol can also alleviate discomfort associated with oral irritations, thereby improving the overall acceptability of buccal products [62].

#### 2.3.2. Results of Viscosity Test

**Figure 2:**
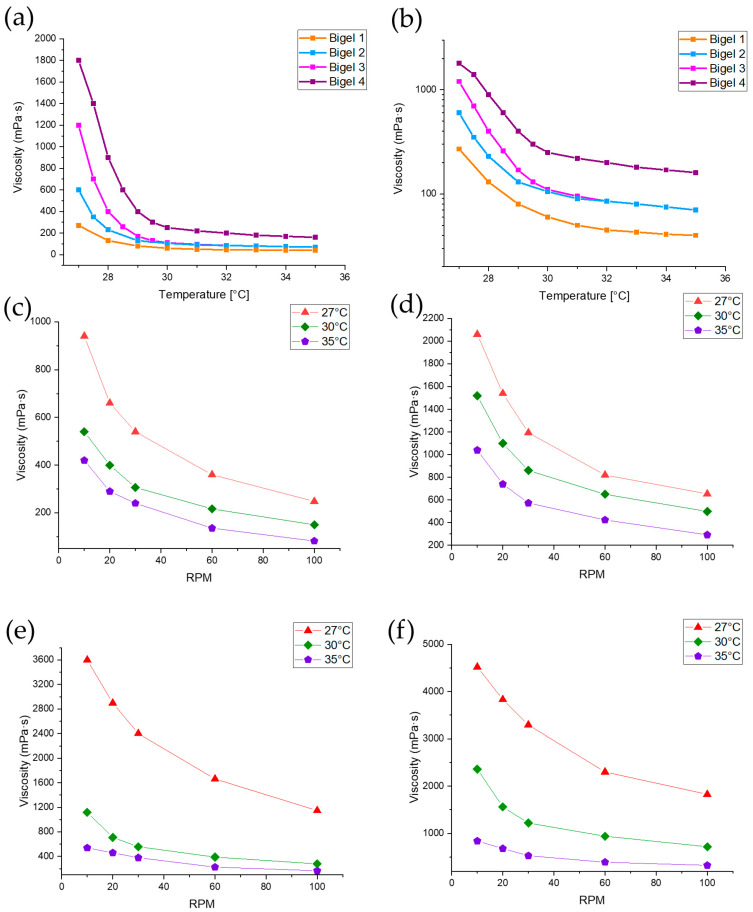
Viscosity of bigels as a function of temperature and velocity (RPM), temperature-dependent viscosity of bigel 1–4 (**a**,**b**), viscosity at different velocities (RPM) and temperatures (27 °C, 30 °C, 35 °C)—bigel 1 (**c**), bigel 2 (**d**), bigel 3 (**e**), bigel 4 (**f**).

Since viscosity is influenced by the crosslinking reaction, experiments on the influence of temperature can help to develop a printing process [63,64]. In the study, the viscosity of the formulations was obtained in the temperature range of 27–35 °C. As shown in Figure 2a,b, all bigel formulations demonstrated a decrease in viscosity with increasing temperature (27 °C to 36 °C), which is characteristic of thermoresponsive materials. The viscosity profile of the four formulations indicates that the higher gelatine content (8% in formulations 3 and 4) results in a significantly higher initial viscosity compared to a formulation with lower gelatine content (5% in formulations 1 and 2). Gelatine forms a gel through the transition of its macromolecules from random coils to triple helices upon cooling, leading to the development of a compact and viscous structure, with notable increases in viscosity observed in gels 3 and 4 [65]. The higher HPMC content (1% in formulations 2 and 4) has the effect of increasing the viscosity of the formulation over the entire temperature range. Viscosity increases more rapidly above 30 °C, which is due to the presence of gelatine in the formulation. Based on the research results, it can be concluded that temperature, gelatine, and HPMC content can strongly influence the viscosity of the formulation. As the formulation is to be used as a 3D printer ink, the formulation of bigel 1 is considered to be the most suitable, as it does not show sudden viscosity changes with temperature but still can create a stable structure after cooling down. Printing parameters can remain unchanged even when the formulation cools down during the process. Figure 2c–f illustrate the influence of rotational velocity (RPM) on viscosity for all bigel formulations at different temperatures (27 °C, 30 °C, and 35 °C). The viscosity analysis of the bigels revealed a shear-thinning behavior, characteristic of non-Newtonian pseudo-plastic systems. The results showed that viscosity decreased with increasing velocity (RPM), indicating structural breakdown under shear stress. Bigel 4, containing 8% gelatin and 1% HPMC, exhibited the highest viscosity values across all velocities, demonstrating enhanced structural stability and resistance to shear forces. Conversely, bigel 1, with 5% gelatin and 0.5% HPMC, showed the lowest viscosity and the steepest decrease with increasing velocity, reflecting a less cohesive gel network. The higher concentration of gelatin improved the initial viscosity, while HPMC contributed to maintaining structural integrity under shear.

Research indicates that as the concentration of gelatin increases, the viscosity of the solution also increases. For instance, Erencia et al. [66] demonstrated that gelatin solutions with higher concentrations exhibited a substantial increase in viscosity over time, particularly in the presence of a high water content and acetic acid, which is indicative of the gelation phenomenon. Temperature control is crucial in the 3D printing process of gelatine, as its viscosity changes significantly with temperature fluctuations. The addition of HPMC as an additional densifying ingredient favorably influences the rheological properties, the viscosity of the print material, and, at the same time, its mucoadhesion properties [67].

#### 2.3.3. Results of In Vitro Dissolution Study

**Figure 3:**
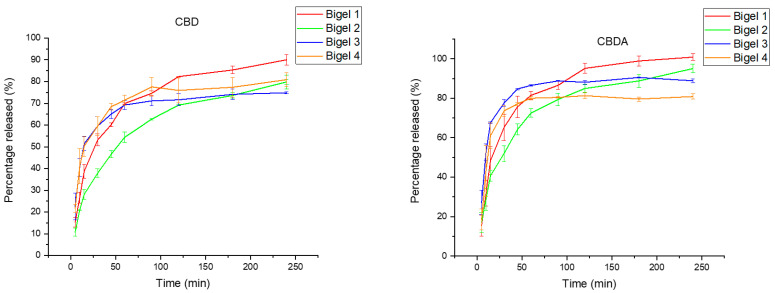
CBDA and CBDA release curves from bigel formulations 1–4.

The study was conducted for the main active compounds present in hemp extract—CBD and CBDA. From the determined curves (Figure 3), it can be concluded that each formulation allows the release of CBD and CBDA in the medium at pH 7.4, mimicking the oral cavity environment. The CBD and CBDA release curves from the four bigels formulations show that the gelatine and HPMC content significantly affects the release rate of the active substances. Bigels with a higher gelatine content (8% in bigels 3 and 4) show faster CBD release, suggesting that gelatine creates a structure that promotes diffusion. Gelatine, being a hydrophilic polymer, rapidly absorbs water, which can lead to an increase in volume and a changing of the bigel structure. Higher concentrations of gelatine may lead to a faster breakdown of the gel matrix, which may facilitate the migration of CBD and CBDA molecules. On the other hand, a higher concentration of HPMC (1% in bigels 2 and 4) slows down the release, especially for CBDA, which may be due to its ability to form a gel barrier that limits particle migration. Finding formulations that enhance the solubility of CBD and CBDA is crucial for increasing their pharmaceutical availability. Many different solutions have been proposed so far. The formation of inclusion complexes with cyclodextrins has been investigated as a method to enhance the solubility and stability of cannabinoid acids like CBDA [68]. Cyclodextrins can encapsulate these compounds, thereby improving their physicochemical properties and making them more bioavailable [68]. In the study by Abdella et al. [69], a hydroxyethyl cellulose (HEC)-based gel containing CBD-NLCs was prepared as the feedstock for pressure-assisted 3D micro-syringe printing, successfully producing buccal films with CBD. These films showed a controlled release profile, with an 84.11 ± 7.02% CBD release over six hours in vitro. Orodispersible tablets were also proposed, e.g., by Vlad et al. [70], achieving 99.3% CBD release in 30 min for pediatric epilepsy and Limpongsa’s team developed directly compressible orally disintegrating tablets of cannabidiol using a liquisolid technique, achieving a dissolution efficiency of 93.5% in ethanol-based formulations [71].

The release profiles of CBD and CBDA from bigel formulations (bigel 1–4) were analyzed using five kinetic models: Zero-Order, First-Order, Higuchi, Hixson–Crowell, and Korsmeyer–Peppas [72]. Each model provided insights into the underlying release mechanisms, and the release constants (*k*_0_, *k_t_*, *k_H_*, *k_HC_*, *k_KP_*) and diffusion exponent were calculated alongside determination coefficients (R^2^) to assess the fit of the models (Table 6 and Table 7). The Zero-Order model, which assumes a constant release rate independent of drug concentration, showed moderate R^2^ values for most bigels, suggesting that it does not fully capture the release mechanisms. Similarly, the First-Order model, which assumes that release depends on the drug concentration remaining, provided slightly better fits for certain bigels, particularly CBDA. However, the lower R^2^ values indicated that the release mechanism might not be purely concentration-dependent.

The Higuchi model, which assumes Fickian diffusion as the primary release mechanism, demonstrated excellent fits for both CBD and CBDA, with R^2^ > 0.82 in bigels 1 and 2, particularly for CBDA formulations [73]. The release constant *k_H_* further supported diffusion-driven release from the gel matrix. The Hixson–Crowell model, which accounts for changes in surface area and particle size during release, showed moderate R^2^ values for some bigels, but it was less suitable than the Higuchi and Korsmeyer–Peppas models. Notably, the *k_HC_* values were low or negative in certain cases, indicating that this model might not adequately describe the release process for these formulations.

The Korsmeyer–Peppas model emerged as the most suitable model for both CBD and CBDA bigels, particularly CBDA, with R^2^ values exceeding 0.95 in some cases. The release constant (*k_KP_*) and the n value (diffusion exponent) consistently indicated a supercase-II transport mechanism, characterized by matrix erosion and swelling effects. This suggests that the release is predominantly driven by the erosion of the gel matrix, with diffusion playing a secondary role. For CBD formulations, *n* values greater than 2 confirmed supercase-II transport, highlighting the importance of matrix dynamics in controlling release. Similarly, for CBDA formulations, both Korsmeyer–Peppas and Higuchi models pointed to matrix erosion as the dominant release mechanism.

In conclusion, the analysis confirmed that the Higuchi and Korsmeyer–Peppas models best describe the release profiles of both CBD and CBDA formulations. The primary release mechanisms involve diffusion and matrix erosion, with supercase-II transport emerging as the dominant mode. While Hixson–Crowell and First-Order models provided additional insights, their lower R^2^ values suggest that they are less robust in capturing the true release dynamics of these systems. These findings underscore the importance of matrix characteristics in drug release and provide a foundation for optimizing formulations to achieve desired release profiles.

### 2.4. 3D Printing Process

**Figure 4:**
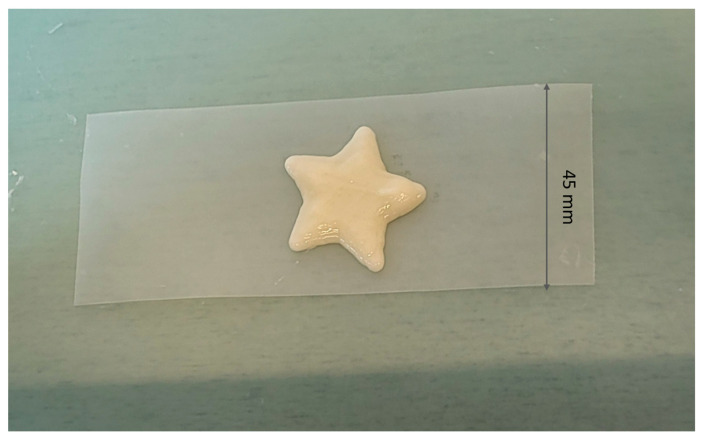
Star-shaped bigel after printing.

The 3D printing of the bigel yielded promising results, with the printed structures maintaining their intended shape and demonstrating high fidelity to the digital model (Figure 4). Importantly, the material’s mass remained consistent across multiple prints, indicating the reproducibility of the process. The bigel exhibited stable rheological properties during printing, allowing for precise deposition and ensuring that the structural integrity was preserved throughout the fabrication process, even despite the application of no temperature control during printing. As bigel 1 showed the least rapid change in properties in viscosity tests, while retaining its structure after time, it could be used in this type of printing. These results are in line with previous studies on similar biomaterials, where bigels were also a suitable material for 3D printing [74,75]. The confirmed mass constancy (1.689 ± 0.001) indicates that 3D printing can be used to personalize the dosage of an active compound in patients who require precise dosing. The average content of CBD is 11.613 mg ± 0.192, and the average content of CBDA is 4.732 mg ± 0.280 in the printout. The proposed method can also be implemented to create delivery systems containing high-THC cannabis extract, as is needed in the treatment with this compound [76]. In the study by Andriotis et al. [77], a cannabis seed oil oleogel structured with 20% *w*/*w* glycerol monostearate was mixed with a 2% *w*/*w* xanthan gum hydrogel in various ratios (0–75% *w*/*w* hydrogel) using a syringe-to-syringe method, creating 3D-printable food inks. This process allowed a consistent mix of oleogel and hydrogel phases, incorporating air uniformly. Printability tests with a standard 3D food printer showed that higher hydrogel ratios negatively impacted print quality, while pure oleogel exhibited superior performance. However, in our study, a formulation with a high hydrogel content also performed well in 3D printing. In the study conducted by Xie et al. [78], bigels containing monoglycerides with an oleogel-to-hydrogel ratio of 7:3 exhibited mechanical properties suitable for 3D printing. This research also incorporated gelatin as a gelling agent in the aqueous phase, alongside different emulsifiers such as PGPR, monoglycerides, and lecithin. Bigels formulated with monoglycerides or lecithin successfully formed homogeneous and stable gel systems. Similarly, Qui et al. [79] identified that a bigel ink with an 80% oleogel fraction was the optimal formulation for 3D printing. Fernandes et al. [74] explored bigels with beeswax as the oleogelling agent and a combination of agar and xanthan gum as hydrogelators. Their findings indicated that bigels containing 10–20% oleogel demonstrated potential as inks for extrusion-based 3D printing. Additionally, Chen et al. [61] confirmed that O/W bigels were more suitable for printing small-area monolayer structural models. Studies carried out by other teams indicate that bigels with different oil phase contents can be successfully printed.

## 3. Conclusions

In conclusion, the successful development of a bigel-based drug delivery system incorporating hemp extract, specifically with known CBD and CBDA content of 11.613 mg ± 0.192 of CBD and 4.732 mg ± 0.280 of CBDA, hyaluronic acid, and menthol marks a significant advancement in personalized medicine. This innovative formulation not only facilitates customizable dosages but also enables controlled release of CBD and CBDA, enhancing therapeutic efficacy and patient compliance. The release profile of CBD and CBDA was evaluated to determine the delivery efficiency of the active ingredient—dissolved active ingredient levels ranged from 74.84% ± 0.50 to 80.87% ± 3.20 for CBD and from 80.84 ± 1.33 to 98.31 ± 1.70 for CBDA depending on the formulation. Bigels offer a compelling dosing option even for pediatric applications, featuring customizable textures, shapes, and flavors that improve palatability, simplify administration, and increase adherence even among younger patients. Additionally, the system demonstrated antibacterial properties against oral pathogens, further expanding its potential applications in oral health. The biphasic structure of bigels allows for the simultaneous encapsulation and delivery of diverse therapeutic agents, making them highly versatile. This platform also paves the way for the incorporation of other therapeutic extracts, broadening its potential in treating various medical conditions. Its adaptability positions the bigel-based system as a promising candidate for future personalized, multi-functional treatments tailored to individual patient needs and improving overall treatment outcomes.

## 4. Materials and Methods

### 4.1. Chemicals and Solvents

Cannabinoid standards solutions, 1 mg/mL in methanol, including CBD, CBDA, CBG, CBN, CBGA, Δ9-THC, Δ8-THC, CBC, THCA (Sigma-Aldrich, Burlington, MA, USA), hemp oil (Skolej, Kościelec, Poland), L-menthol (Sigma-Aldrich), triple hyaluronic acid 1.5% (aqueous solution with 0.5% sodium hyaluronate (0.85–1.15 MDa), 0.5% (<500 kDa), and 0.5% (<10 kDa)), gelatin 20 mesh particle size (Chempur, Karlsruhe, Germany), (Hydroxypropyl)methyl cellulose M_W_ = ~86 kDa, viscosity 2600–5600 cP, 2% in H_2_O (20 °C) (Sigma Aldrich), phosphate buffered saline tablets (Fisher Scientific, Pittsburgh, PSA, USA), Tween 80 (Merck KGaA, Darmstadt, Germany), L-α-lecithin (EMD Millipore Corp., Darmstadt, Germany), acetonitrile for HPLC (VWR Chemicals, Radnor, PA, USA), trifluoroacetic acid (Honeywell, Albany, NY, USA); Hemp strains obtained from local markets: Silver Haze (Swiss Hemp, Biel/Bienne, Switzerland), CBD < 10%, THC < 0.2%, White Widow (Swiss Hemp), THC < 0.2%, Red Cherry (Swiss Hemp), CBD < 10%, THC < 0.2%, Pineapple Express (Swiss Hemp), CBD < 10%, THC < 0.2%, AK47 (Swiss Hemp), CBD < 10%, THC < 0.3%, Amnesia (Swiss Hemp; CBD < 10%, THC < 0.2%, Black Widow (Creathink, Nanjing, China). Mueller-Hinton broth was obtained from Graso (Poland). MTT (3-(4,5-dimethyl-2-thiazolyl)-2,5-diphenyl-2H-tetrazolium bromide) was purchased from Sigma-Aldrich.

### 4.2. Raw Material for Testing

In order to find the cannabis with the highest cannabinoid content, seven strains were subjected to extraction of seven strains:

1. Silver Haze
2. White Widow
3. Red Cherry
4. Pineapple Express
5. AK47
6. Amnesia
7. Black Widow

### 4.3. Extraction of Raw Material Using Supercritical CO_2_

The extraction process with scCO_2_ was carried out using a SFT-120 apparatus (Supercritical Fluid Technologies, Inc., Newark, DE, USA) at a pressure of 6000 PSI and a temperature of 50 °C with 250 mL of CO_2_. The dried material of the above-mentioned varieties were crushed and weighed, then loaded into the 5 mL vessel of the extraction apparatus. The weight of the raw material in the dish was approximately 6.0 g and was accurately determined for each sample for further calculations. The extracts were then suspended in 5 mL of methanol and further analyzed to choose variety with the highest content of cannabinoids. The extraction of the strain selected for further study was carried out under the same conditions but repeated 5 times, each time with a fresh portion of raw material. Obtained extracts were combined and dissolved in 150 mL of ethanol (73.1 mg of pure extract/mL). The extract obtained by this technique contains many waxes, so the next step was to carry out a purification process by winterization. Winterization was carried out by placing the extract in a freezer at −18 °C for a period of 48 h. This was followed by filtration under reduced pressure, leaving the waxy impurities on the filter while the extract became clearer. The procedure was repeated twice.

### 4.4. Analysis of the Content of Active Compounds Using HPLC

Analysis of the cannabinoid profile of the extracts was performed by ultra-high-performance liquid chromatography using a validated method with a diode array detector (HPLC-DAD) (Shimadzu Corporation, Kyoto, Japan). The determination was performed according to the methodology [80]. The determination was carried out using a CORTECS Shield RP18, 2.7 µm; 150 mm × 4.6 mm chromatography column. 0.1% trifluoroacetic acid (41%) and acetonitrile (41:59, *v*/*v*) were used as the mobile phase. The flow rate was set at 2.0 mL/min, and the column temperature was set at 35 °C. The injection volume was 10.0 µL, the detection wavelength was 228 nm, and the analysis time was 50 min. The retention time is shown in the table below (Table 8).

### 4.5. Analysis of the Content of Active Compounds Using Gas Chromatography (GC–MS)

Fifty µL of extract was taken, and 2.2 mL of absolute ethanol was added and mixed thoroughly. The whole was filtered through a 0.20 µm syringe filter and subjected to GC–MS analysis. A sample volume of 1 µL was injected into the column. The chromatograph was equipped with a VF-5ms silica column (30 m × 0.25 mm × 0.39), df = 0.25 Crawford Scientific. The electron energy was 70 eV, and the ion source was at 200 °C. Helium was used as the carrier gas at a flow rate of 1 mL/min. Identification of the compounds was based on a comparison of their retention times as well as mass spectra with standards from NIST.

### 4.6. Analysis of Antimicrobial Properties

The minimum inhibitory concentration (MIC) of the cannabis extract was determined using the microdilution method with 96-well plates (Nest Scientific Biotechnology, Jiangsu, China). Details are described in previous articles [81]. The activity was tested against strains of *Staphylococcus aureus*, *Pseudomonas aeruginosa*, *Candida albicans*, *Streptococcus mutans* ATCC 25175, *Porphyromonas gingivalis* ATCC 33277, *Fusobacterium nucleatum* ATCC 25586. The bacteria *S. aureus*, *P. aeruginosa*, and *S. mutans* were cultured in tryptone soy broth (Graso Biotech, Starogard Gdański, Poland), the yeast *C. albicans* was cultured in Sabouraud broth (Graso Biotech, Starogard Gdański, Poland), and the anaerobes *P. gingivalis* and *F. nucleatum* were cultured in Schaedler broth (Graso Biotech, Starogard Gdański, Poland). Serial dilutions of each antiseptic were prepared, starting from a concentration of 73 mg/mL. The plates were incubated at 36 °C for 24–48 h. Additionally, octenidine dihydrochloride (Schülke & Mayr, Norderstedt, Germany) was used as a control substance, starting at a concentration of 10 µg/mL.

### 4.7. Bigel Preparation

To prepare bigels with the desired properties, ingredients were carefully selected in various proportions to achieve the desired effects. The substances used in each formulation tested include the following:

- Purified water—aqueous phase
- Hemp oil—oil phase (carrier for hemp extract)
- Gelatine—hydrogelator
- Lecithin—oleogelator
- Hydroxypropyl methyl cellulose (HPMC)—adhesion enhancer
- 1.5% Hyaluronic acid salt solution—moisturizer
- Menthol—taste enhancer

Formulations were obtained with the compositions shown in Table 5 in Section 2.3. For each formulation, the execution followed the same process. In a glass beaker, hemp oil was heated to 80 °C, and then lecithin and menthol were added and mixed with a dipstick. Water in the beaker was then heated to 80 °C, gelatine and HPMC were added and mixed until dissolved, then hyaluronic acid was added and mixed again. The 5-fold concentrated hemp extract was added to the cooled oil phase and mixed. The two phases were combined and mixed on a magnetic stirrer at low speed for one minute. Homogenization of the bigel was carried out for one minute at 1500 rpm. The formulations are shown in Table 5. Formulations were put into glass containers and cooled to room temperature (RT, 25 °C) for 24 h forming bigels. The formation of bigels was confirmed by the inverted tube method [4].

### 4.8. Viscosity Test

Analysis was performed using a Rotating Disc Viscometer DV2T-RV Extra5 (Ametek Brookfield, Middleboro, MA, USA) equipped with a temperature probe and an RV05 spindle system. The substance was initially heated and then cooled over time, with simultaneous testing performed as the temperature decreased in 1 °C increments. The analysis was conducted in the same beaker with a diameter of 8.25 cm. This measurement corresponds to apparent viscosity, as it reflects the flow behavior of the substance under the applied conditions.

### 4.9. In Vitro Dissolution Study

A release study was conducted in simulated saliva to select the formulation with the highest pharmaceutical availability among the four proposed formulations. Approximately 1.5 g of each formulation was placed in a saliva-simulating fluid, which was phosphate buffer pH 7.4 with 1% Tween 80 as surfactant. The test was carried out using a 708-DS Dissolution Apparatus (Agilent Technologies) with a basket rotation speed of 50 RPM. Dissolution samples were taken at appropriate time points and were filtered through 0.22 μm nylon membrane syringe filters.

To determine the release mechanisms of CBD and CBDA from bigel formulations, the release data were fitted to five mathematical kinetic models: Zero-Order, First-Order, Higuchi, Hixson–Crowell, and Korsmeyer–Peppas [72]. These models were selected based on their ability to describe various drug release mechanisms, including diffusion, erosion, and their combinations. Linear regression was applied to each dataset for all models. The release constants were obtained from the slopes or intercepts of the fitted lines, depending on the model. The fit of each model was evaluated using the correlation coefficient (R^2^), with the highest R^2^ value indicating the best-fitting model and the most probable release mechanism. The models are represented by the following equations:

(1) $$F_{t}=k_{0}\times t$$

(2) $$F_{t}=1-e^{-k_{1}t}$$

(3) $$F_{t}=k_{H}\sqrt{t}$$

(4) $$\sqrt[{3}]{F_{0}}-\sqrt[{3}]{F_{t}}={k_{HC}}^{t}$$

(5) $$F_{t}={kt}^{n}$$

$F_{t}$—the fraction of CBD/CBDA released in time

*k*_0_, *k*_1_, *k_H_*, *k_HC_*, *k_KP_*—release constants of particular kinetic models

*n*—the diffusion exponent

For the Korsmeyer–Peppas model, the *n* value was calculated to classify the release mechanism [82]:

- *n* ≤ 0.45: Fickian diffusion
- 0.45 < *n* < 0.89: Anomalous transport
- *n* = 0.89: Erosion-controlled (Case II transport)
- *n* > 0.89: Supercase-II transport

### 4.10. 3D Printing

The printing process was carried out using a 3D printer, model Moore 2 Pro (Shenzen Tronxy Technology, Shenzhen, Guangdong, China) using a 1.4 mm nozzle. Using computer software (Audodesk’s Tinkecad, online access: https://www.tinkercad.com/ accessed on 1 October 2024), a design of the formula shape was created and then transferred to the printer system (Ultimaker Cura 4.13.1). The design is a single-layer star shape with dimensions of 30 mm × 35 mm × 2 mm. The print speed was set to 10 mm/s, and the nozzle height to 4 mm. The bigel was heated to 35 °C before printing and allowed to cool and crosslink for 24 h after printing. The average CBD and CBDA content of the printed products was determined by dissolving them in methanol, sonification, and determination by the HPLC method.

## Figures and Tables

**Table 1:** Cannabinoids content in different strains of *Cannabis sativa* L.

Variety	CBD * (mg/g)	CBDA (mg/g)	CBG (mg/g)	CBN (mg/g)	CBGA (mg/g)	Δ^9^-THC (mg/g)	Δ^8^-THC (mg/g)	CBC (mg/g)	THCA (mg/g)	Total Cannabinoids (mg/g)
Silver Haze	26.684 ± 1.067	5.616 ± 0.112	0.434 ± 0.017	0.148 ± 0.006	0.061 ± 0.002	0.641 ± 0.025	0.035 ± 0.001	0.902 ± 0.036	0.000 ± 0.000	34.520 ± 0.690
White Widow	16.435 ± 0.657	5.828 ± 0.117	0.276 ± 0.011	0.082 ± 0.003	0.085 ± 0.003	0.558 ± 0.022	0.007 ± 0.000	0.492 ± 0.020	0.000 ± 0.000	23.761 ± 0.475
Red Cherry	10.731 ± 0.429	3.811 ± 0.076	0.063 ± 0.003	0.065 ± 0.003	0.034 ± 0.001	0.318 ± 0.013	0.008 ± 0.000	0.326 ± 0.013	0.023 ± 0.001	15.379 ± 0.307
Pineapple Express	9.248 ± 0.370	3.350 ± 0.067	0.123 ± 0.005	0.054 ± 0.002	0.057 ± 0.002	0.257 ± 0.010	0.012 ± 0.000	0.346 ± 0.014	0.017 ± 0.001	13.464 ± 0.269
AK47	15.375 ± 0.615	3.860 ± 0.077	0.259 ± 0.010	0.061 ± 0.002	0.061 ± 0.002	0.393 ± 0.016	0.020 ± 0.001	0.650 ± 0.026	0.014 ± 0.001	20.694 ± 0.415
Amnesia	12.610 ± 0.504	7.399 ± 0.148	0.244 ± 0.010	0.032 ± 0.001	0.198 ± 0.008	0.522 ± 0.021	0.000 ± 0.000	0.453 ± 0.018	0.161 ± 0.003	21.618 ± 0.432
Black Widow	23.490 ± 0.940	15.355 ± 0.307	0.560 ± 0.022	0.103 ± 0.004	0.302 ± 0.012	0.931 ± 0.037	0.005 ± 0.000	0.929 ± 0.037	0.202 ± 0.004	41.877 ± 0.837

* Abbreviations used in the table: CBD: cannabidiol, CBDA: cannabidiolic acid, CBG: cannabigerol, CBN: cannabinol, CBGA: cannabigerolic acid, Δ9-THC: delta-9-tetrahydrocannabinol, Δ8-THC: delta-8-tetrahydrocannabinol, CBC: cannabichromene, THCA: tetrahydrocannabinolic acid.

**Table 2:** Content of cannabinoids in extract assessed by HPLC method.

Cannabinoid	Content (mg/mL)
CBD	164.738 ± 10.088
CBDA	68.756 ± 5.776
CBG	3.489 ± 0.305
CBN	0.690 ± 0.038
CBGA	0.619 ± 0.026
∆^9^-THC	5.487 ± 0.443
∆^8^-THC	16.273 ± 0.921
CBC	0.733 ± 0.132
THCA	-

Abbreviations used in the table: CBD: cannabidiol, CBDA: cannabidiolic acid, CBG: cannabigerol, CBN: cannabinol, CBGA: cannabigerolic acid, Δ9-THC: delta-9-tetrahydrocannabinol, Δ8-THC: delta-8-tetrahydrocannabinol, CBC: cannabichromene, THCA: tetrahydrocannabinolic acid.

**Table 3:** Content of active compounds assed by GC–MS method.

Lp.	Retention Time (min)	Percentage of Total (%)	Compound
1	5.761	0.324	cis-5.8.11.14.17-Eicosapentaenoic acid
2	13.524	2.548	Caryophyllene
3	13.624	0.191	7-epi-cis-sesquisabinene hydrate
4	13.989	0.826	Humulene
5	14.574	0.212	α-acorenol
6	15.005	0.191	ß-Guaiene
7	15.068	0.267	(4aR-trans)-decahydro-4a-methyl-1-methylene-7-(1-methylethylidene)-Naphthalene
8	15.582	0.249	(3ß.5α)-2-methylene-Cholestan-3-ol
9	16.063	0.304	8-epi-γ-eudesmol
10	16.520	0.572	α-acorenol
11	16.702	0.286	7-epi-cis-sesquisabinene hydrate
12	23.748	93.848	Cannabidiol

**Table 4:** Antimicrobial activity of the hemp extract and control octenidine.

Pathogen	Hemp Extract	Octenidine
	MIC (mg/mL)	MIC (µg/mL)
*Staphylococcus aureus*	73 ± 0.0	1.25 ± 0.0
*Pseudomonas aeruginosa*	36.5 ± 0.0	1.88 ± 0.9
*Streptococcus mutans* *ATCC 25175*	4.6 ± 0.0	0.6 ± 0.0
*Candida albicans*	36.5 ± 0.0	2.5 ± 0.0
*Porphyromonas gingivalis* *ATCC 33277*	36.5 ± 0.0	0.6 ± 0.0
*Fusobacterium nucleatum* *ATCC 25586*	36.5 ± 0.0	0.6 ± 0.0

**Table 5:** Quantitative composition of bigels.

Ingredient	Bigel 1 (%)	Bigel 2 (%)	Bigel 3 (%)	Bigel 4 (%)
Water	72.4	71.9	69.4	68.9
Oil	10	10	10	10
Lecithin	2	2	2	2
Extract	5	5	5	5
Gelatin	5	5	8	8
1.5% Hyaluronic acid salt solution	5	5	5	5
Hydroxypropyl methyl cellulose	0.5	1	0.5	1
Menthol	0.1	0.1	0.1	0.1

**Table 6:** Parameters of kinetic release models suggesting the mechanism of CBD liberation from bigel formulation.

	Zero-Order		First-Order		Higuchi		Hixson–Crowell		Korsmeyer–Peppas		
	*k*_0_	R^2^	*k*_1_	R^2^	*k_H_*	R^2^	*k_HC_*	R^2^	*k_KP_*	*n*	R^2^
Bigel 1	0.278	0.725	0.005	0.548	5.457	0.888	−0.009	0.864	0.435	2.315	0.921
Bigel 2	0.198	0.865	0.004	0.764	4.238	0.952	−0.006	0.932	0.37	2.404	0.974
Bigel 3	0.156	0.517	0.003	0.403	3.236	0.706	−0.004	0.598	0.264	3.04	0.804
Bigel 4	0.182	0.554	0.003	0.428	3.753	0.743	−0.005	0.649	0.294	2.959	0.827

**Table 7:** Parameters of kinetic release models suggesting the mechanism of CBDA liberation from bigel formulation.

	Zero-Order		First-Order		Higuchi		Hixson–Crowell		Korsmeyer–Peppas		
	*k*_0_	R^2^	*k*_1_	R^2^	*k_H_*	R^2^	*k_HC_*	R^2^	*k_KP_*	*n*	R^2^
Bigel 1	0.295	0.641	0.005	0.466	5.940	0.823	−0.014	0.868	0.439	2.456	0.865
Bigel 2	0.291	0.741	0.005	0.570	5.696	0.899	−0.010	0.905	0.424	2.410	0.934
Bigel 3	0.167	0.404	0.003	0.320	3.597	0.595	−0.006	0.511	0.253	3.310	0.723
Bigel 4	0.159	0.355	0.003	0.277	3.488	0.543	−0.005	0.414	0.302	2.992	0.675

**Table 8:** Cannabinoid retention time.

Cannabinoid	Retention Time [min]
CBD	5.84
CBDA	6.42
CBG	6.82
CBN	8.72
CBGA	9.22
Δ^9^-THC	10.27
CBC	14.57
THCA	16.31

## Data Availability

The data presented in this study will be openly available in the Ze-nodo repository.
